# Loot

**Published:** 1868-03-01

**Authors:** 


### LOOT.

### *Iodine and Carbolic Acid.*

Dr. Percy Boulton, to remedy the inconvenience attending the external application of iodine and its preparations, has adopted the method of adding a few drops of carbolic acid to the iodine solution to be employed. The formula is as follows: Compound tincture of iodine, 3 grms; pure liquid carbolic acid, 6 drops; glycerine, 30 grms; distilled water, 150 grms. This carbolate of iodine is not perfectly colorless, so that it may be applied with impunity; and it is not only one of the most powerful antiseptics we possess, but is intrinsically a more efficacious agent than iodine alone. In the form of injections, gargles, and lotions, for sore throat, ozœna, abscess in the ear, etc., this preparation is a sovereign remedy.—*Extract from a letter in the Journal des Connaissance Médicales.—Med. Gazette.*

### *Gentian Root as a Dilator.*

Professor Winckel, in Rostock, recommends (Deut. Klinik, 1867) the radix gentianæ rubræ as a new, simple and cheap means of dilatation for surgical and gynecological purposes. His attention was first directed thereto by an article of John Jacob Hacberl, published in 1834, in which the author states that, having operated for atresia uteri, and desiring to keep open the orifice made by the trocar, he introduced a good firm plug of radix gentianæ, and that on the following day he found no small difficulty in withdrawing the same, which had increased to twice its former size. According to Dr. Winckel's observations, the gentian root has the following advantages over laminaria: 1st, its cheapness, the ease with which it can be obtained, and the fact that the physician can so easily cut plugs and bougies of any size to suit his requirements. 2nd, its somewhat smaller power of absorption, as compared with laminaria, is compensated by our being able to obtain larger pieces of it (one and a half to two inches in diameter), so that it can be used for the dilatation of openings already too large for laminaria. 3rd, the fact of its remaining free from smell constitutes an immense advantage, for even laminaria, though in a much less degree than sponge-tents, often becomes quite fœtid. The radix gentianæ may, therefore, be used with special advantage in strictures of the vulva, vagina, and uterus; for tamponing the uterus in smaller hæmorrhages, for the induction of abortion, for dilatation after operation for atresia of the genital organs. Whether it is also applicable to stricture of the urethra, to affections of the lachrymal ducts, etc., remains to be seen.—*Med. Record, from All. Med. C. C. Ztg.*, 1867.—*Medical Gazette.*

Dr. Maisonneuve has also an apparatus contrived by him for the purpose of removing the cause which most frequently occasions death after amputation. He maintains that the feverish symptoms, which appear in a variety of shapes, in all cases of wounds, and constitute the chief danger of surgical operations, are invariably the consequence of poisonous action. This, in his opinion, is owing to the fact that the liquids exuding from sores are deprived of vitality by contact with the atmosphere, then undergo a process of putrefaction, and are thus converted into dangerous poisons. He thence concluded that if he could prevent these dead liquids from becoming putrefied at the surface of the solution of continuity, the amputation of limbs might be effected without in the slightest degree endangering the patient's life. In order to obtain this result, Dr. Maisonneuve conceived the idea of subjecting the stump of the amputated limb to a constant process of suction, whereby the liquids might be abstracted as soon as they appear. For this purpose he has contrived a sort of india-rubber hood, which may be put upon the stump after preliminary dressing; that is, after the ligature of the blood vessels has been duly performed, the wound properly washed, and the borders kept together by means of straps of diachylon plaster, but so as not to prevent the exudation of the dangerous liquids. The hood, which, at its orifice, firmly embraces the stump, is provided at the opposite extremity with an india-rubber tube communicating with a tubulated balloon, which in its turn communicates with a suction pump. In this way, owing to the vacuum made, the obnoxious liquids are carried off, and the hood, collapsing on the stump, keeps the borders of the wound firmly pressed together.—*Cor. Leavenworth Herald.*

